# Supplementary material for: Nanoconfinement facilitates reactions of carbon dioxide in supercritical water
Source: Nat Commun. 2022 Oct 8;13:5932. doi: 10.1038/s41467-022-33696-w (PMC9547913; doi:10.1038/s41467-022-33696-w)
Supplement: Supplementary file 1 — Supplementary Information [file 41467_2022_33696_MOESM1_ESM.pdf]

Supplementary Information for: Nanoconfinement Facilitates Reactions of Carbon Dioxide in Supercritical Water

Nore Stolte,[1] Rui Hou,[1,2] and Ding Pan[1,3,2,*]

[1]*Department of Physics, Hong Kong University of Science and Technology, Hong Kong, China*[†]

[2]*HKUST Shenzhen-Hong Kong Collaborative Innovation Research Institute, Shenzhen, China*

[3]*Department of Chemistry, Hong Kong University of Science and Technology, Hong Kong, China*

[*] dingpan@ust.hk

[†] Present address: Lehrstuhl für Theoretische Chemie, Ruhr-Universität Bochum, 44780 Bochum, Germany

SUPPLEMENTARY METHODS

Because the PBE exchange-correlation functional cannot calculate the van der Waals interactions between graphene sheets and solutions well [1], we modeled graphene in contact with solutions by defining a potential that acts on carbon and oxygen atoms in solutions as a function of the perpendicular distance d between graphene and atoms. A benefit of using a model potential is that there is no surface chemistry or charge transfer between the model potential and solutions, so by comparing it with the more realistic stishovite-confined solutions, we can distinguish the effects of spatial confinement and surface chemistry. Using a model potential to create nanoconfinement for aqueous solutions has been adopted in many previous studies (e.g., [2–4]). The graphene-oxygen interaction was fitted to the diffusion Monte Carlo calculations for water molecules with the two-legged configuration adsorbed on the graphene sheet [5]; we put the molecular force on oxygen atoms. The interaction has the Morse potential shape (see Supplementary Fig. 1):

$$E_b^{\mathrm{O}}(d) = D_e^{\mathrm{O}} \left[\left(1 - e^{-a^{\mathrm{O}}(d - d_0^{\mathrm{O}})} \right)^2 - 1 \right], \tag{1}$$

where $D_e^{\mathrm{O}} = 9.55185$ kJ mol^{-1}, $a^{\mathrm{O}} = 1.34725$ Å^{-1}, and $d_0^{\mathrm{O}} = 3.37265$ Å [5].

We tried several force potentials to fit the graphene-CO_2 interaction using the optB86-vdW functional reported in [6] (see Supplementary Fig. 1), and found that the Morse potential best reproduces the interaction,

$$E_b^{\mathrm{CO_2}}(d) = D_e^{\mathrm{CO_2}} \left[\left(1 - e^{-a^{\mathrm{CO_2}}(d - d_0^{\mathrm{CO_2}})} \right)^2 - 1 \right], \tag{2}$$

where $D_e^{\mathrm{CO_2}} = 21.50492$ kJ mol^{-1}, $a^{\mathrm{CO_2}} = 1.18449$ Å^{-1}, and $d_0^{\mathrm{CO_2}} = 3.25282$ Å. The graphene-carbon interaction was then obtained by

$$E_b^{\mathrm{C}} = E_b^{\mathrm{CO_2}} - 2E_b^{\mathrm{O}}. \tag{3}$$

However, this gives a nonphysical attractive potential at short distances (<3 Å), as shown in Supplementary Fig. 1(b). To correct this, we added the following repulsive term to the

graphene-carbon interaction:

$$E_b^{\mathrm{rep}} = D_e^{\mathrm{rep}} e^{-2a^{\mathrm{rep}}\left(d-d_0^{\mathrm{rep}}\right)}, \quad (4)$$

where $D_e^{\mathrm{rep}} = 18.69565$ kJ mol^{-1}, $a^{\mathrm{rep}} = 26.09666$ Å^{-1}, and $d_0^{\mathrm{rep}} = 2.56637$ Å. The overall graphene-carbon interaction is

$$E_b^{\mathrm{C}}(d) = D_e^{\mathrm{CO_2}}\left[\left(1-e^{-a^{\mathrm{CO_2}}(d-d_0^{\mathrm{CO_2}})}\right)^2-1\right]-2D_e^{\mathrm{O}}\left[\left(1-e^{-a^{\mathrm{O}}(d-d_0^{\mathrm{O}})}\right)^2-1\right]+D_e^{\mathrm{rep}}e^{-2a^{\mathrm{rep}}(d-d_0^{\mathrm{rep}})}, \quad (5)$$

as shown in Supplementary Fig. 1(b). There is no interaction between graphene and hydrogen atoms in our simulations. We implemented the interaction Eqs. (1,5) in the Qbox code.

To study graphene-confined solutions, we modeled two model graphene surfaces in the xy plane. Water and CO_2 molecules were inserted between these sheets. There is 7 Å thick vacuum, so the confined solution is at least 10 Å away from its replica with periodic boundary conditions. For the convenience of comparison, the x- and y-dimensions of the unit cell are the same as those with stishovite confinement . The simulation details are shown in Supplementary Table I. At ~10 GPa and 1000 K, we also doubled the number of molecules in the unit cell and added the van der Waals corrections [7] to test our computational setups. We used the SG15 Optimized Norm-Conserving Vanderbilt (ONCV) pseudopotentials with a plane-wave cutoff of 65 Ry to speed up calculations [8, 9]. Supplementary Fig. 4 and Supplementary Table IV show that our simulation sizes in Supplementary Table I are big enough, and the van der Waals corrections change the chemical speciation little.

We calculated the lateral pressure in the simulation cell: $P_\parallel = \left(\sigma'_{xx} + \sigma'_{yy}\right)/2$. The diagonal elements of the computed stress tensor, σ_{xx} and σ_{yy}, are modified to account for the vacuum in the unit cell according to $\sigma'_i = \sigma_i\left(L_z/h_z\right)$, where L_z is the z-dimension of the unit cell, and h_z is the distance between graphene sheets. This method was used in previous studies [3].

For stishovite-confined solutions, the stishovite slab is made by three stoichiometric layers

of SiO_2 exposing the low-energy (100) surface [10] to solutions, as shown in Supplementary Fig. 2. Each SiO_2 layer contains 8 SiO_2 formula units, so there are 24 silicon atoms and 48 oxygen atoms in the unit cell. In the NVT simulations, we only fixed the positions of silicon atoms in the middle layer of the stishovite slab. Supplementary Table I summarizes the simulation setups.

To establish whether solutions are acidic or basic, acidity can be quantified using

$$f = \mathrm{pH} - \mathrm{pOH} = -\log_{10}\left(\frac{[\mathrm{H_3O^+}]}{[\mathrm{OH^-}]}\right) = -\log_{10}\left(\frac{N_{\mathrm{H_3O^+}}}{N_{\mathrm{OH^-}}}\right), \tag{6}$$

where $N_{\mathrm{H_3O^+}}$ and $N_{\mathrm{OH^-}}$ are the average numbers of the H_3O^+ and OH^- ions, respectively, in one unit cell per each AIMD snapshot as shown in Supplementary Table VII. For a neutral solution, $f = 0$, while $f > 0$ indicates the solution is basic and $f < 0$ for an acidic solution.

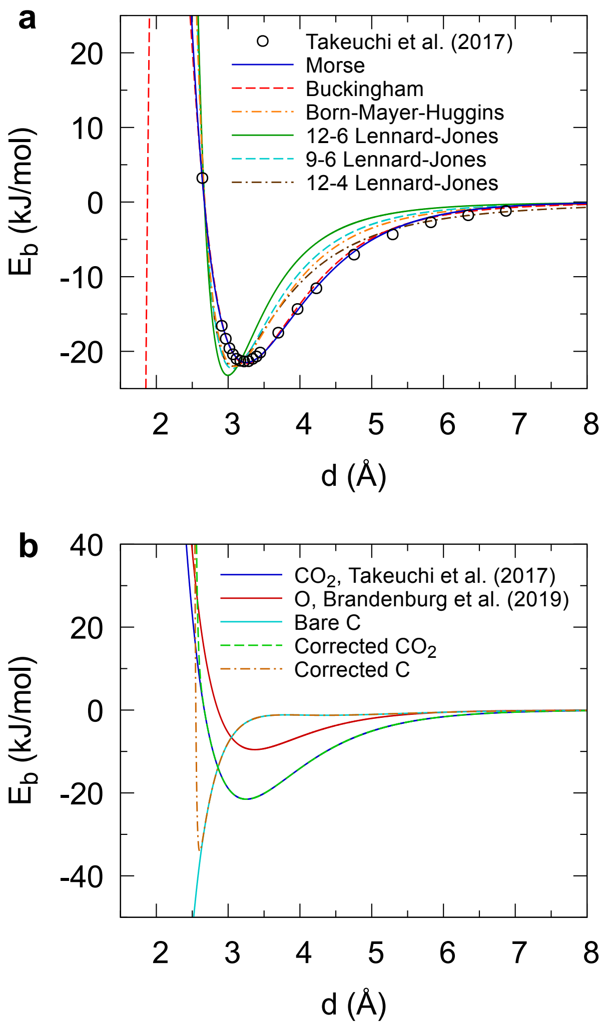

Supplementary Fig. 1. Graphene-carbon and graphene-oxygen interactions. d is the distance between graphene and atoms. (**a**) Several force potentials were fitted to the data from the study on the graphene-CO_2 interaction by Takeuchi et al. [6]. The Morse potential is given by Eq. 2. (**b**) The graphene-oxygen interaction (i.e., graphene-H_2O interaction) is from Brandenburg et al.'s study [5]. The resulting bare graphene-carbon (Bare C) interaction is given by Eq. 3. The graphene-carbon and graphene-CO_2 interactions after adding the repulsive term (Eq. 4) are shown by the dot-dashed (Corrected C) and dashed (Corrected CO_2) lines, respectively.

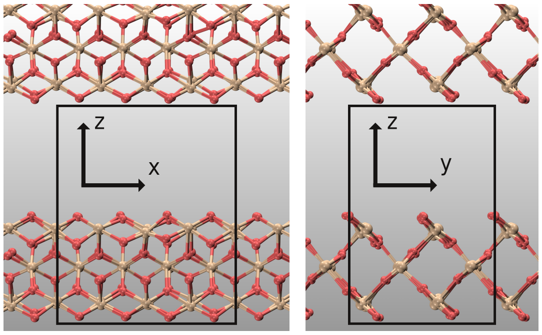

Supplementary Fig. 2. Unit cell with the stishovite confinement. The stishovite slab consists of three stoichiometric layers of SiO_2. The empty space was occupied by the fluid in our simulations. Oxygen atoms in SiO_2 are represented by pink balls, and silicon atoms are represented by yellow balls.

Supplementary Table I. Details of the AIMD simulations. x(CO_2) is the initial mole fraction of CO_2(aq). N_{CO_2}, N_{H_2O}, and N_{SiO_2} are the initial number of CO_2, H_2O, and SiO_2, respectively, in the unit cell. h_z is the distance between two confining surfaces. $P_{\|}$ is the lateral pressure: $P_{\|} = \left(\sigma'_{xx} + \sigma'_{yy}\right)/2$. $P_{\perp}$ is the pressure along the z direction, $P_{\perp} = \sigma_{zz}$. t is the total simulation time after 20 ps equilibration. Bulk results are taken from Ref. 11. The uncertainties are standard deviations.

Confinement	x(CO_2)	N_{CO_2}	N_{H_2O}	N_{SiO_2}	T (K)	h_z (Å)	P (GPa)	ρ (kg m^{-3})	t (ps)
Graphene	0.185	5	22	-	1000	9.0	$P_{\|} = 10.9 \pm 1.4$	1707	180
Graphene	0.185	5	22	-	1400	9.2	$P_{\|} = 9.4 \pm 1.5$	1622	180
SiO_2	0.185	5	22	24	1000	6.9	$P_{\perp} = 9.5 \pm 2.0$	-	480
SiO_2	0.185	5	22	24	1400	7.1	$P_{\perp} = 9.8 \pm 2.2$	-	280
Bulk	0.185	10	44	-	1000	-	P $= 9.4 \pm 1.2$	1757	280
Bulk	0.032	2	60	-	1400	-	P $= 10.4 \pm 1.2$	1545	180
Bulk	0.143	8	48	-	1400	-	P $= 9.2 \pm 1.3$	1613	180
Bulk	0.333	16	32	-	1400	-	P $= 9.0 \pm 1.3$	1702	180

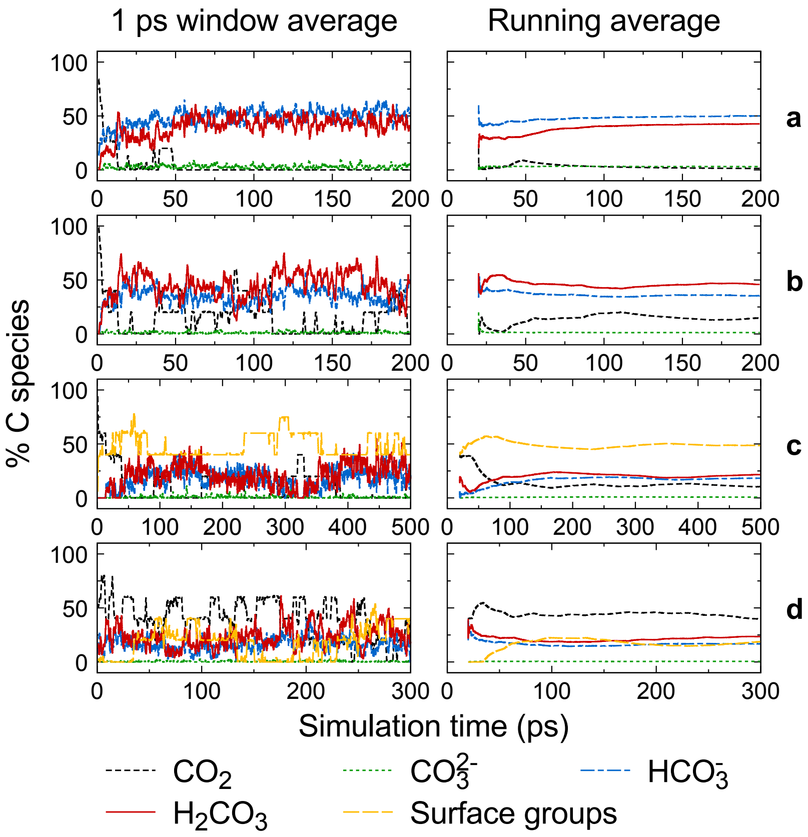

Supplementary Fig. 3. Mole percents of carbon species as functions of simulation time in AIMD simulations. The initial mole fraction of CO_2(aq) is 0.185, and the pressure is ∼10 GPa. Left panel: the 1 ps window average. Right panel: the running average excluding the first 20 ps equilibration. (**a**) 1000 K, graphene confinement. (**b**) 1400 K, graphene confinement. (**c**) 1000 K, stishovite confinement. (**d**) 1400 K, stishovite confinement.

Supplementary Table II. Equilibrium mole percents of carbon species in total dissolved carbon in solutions. The initial mole fraction of CO_2(aq) is 0.185. The pressure is $\sim$10 GPa, and the temperature is 1000 K. Bulk values are taken from Ref. 11.

Confinement	Bulk	Graphene	Stishovite
CO_2	15.1 ± 2.0	1.3 ± 0.9	10.5 ± 2.3
CO_3^{2-}	1.1 ± 0.0	3.1 ± 0.1	0.7 ± 0.1
HCO_3^-	35.9 ± 0.7	50.0 ± 1.0	18.2 ± 1.8
H_2CO_3	46.8 ± 1.5	42.7 ± 1.7	21.6 ± 2.7
$H_3CO_3^+$	0.8 ± 0.1	1.3 ± 0.1	0.5 ± 0.1
Pyrocarbonate	0.3 ± 0.1	1.6 ± 0.8	0.0
Surface groups	-	-	48.5 ± 2.1

Supplementary Table III. Equilibrium mole percents of carbon species in total dissolved carbon in solutions. The initial mole fraction of CO_2(aq) is 0.185. The pressure is ∼10 GPa, and the temperature is 1400 K. The bulk values are obtained by interpolating the results for x(CO_2) = 0.032, 0.143 and 0.333 from Ref. 11 with cubic splines.

Confinement	Bulk	Graphene	SiO_2
CO_2	58.8 ± 2.0	14.5 ± 3.2	39.8 ± 3.6
CO_3^{2-}	0.1 ± 0.0	1.3 ± 0.1	0.5 ± 0.1
HCO_3^-	16.8 ± 0.7	35.5 ± 1.0	16.6 ± 1.2
H_2CO_3	23.6 ± 1.4	45.9 ± 2.3	23.3 ± 1.7
$H_3CO_3^+$	0.3 ± 0.1	2.0 ± 0.6	0.6 ± 0.0
Pyrocarbonate	0.4 ± 0.1	0.7 ± 0.3	0.2 ± 0.1
Surface groups	-	-	19.1 ± 3.1

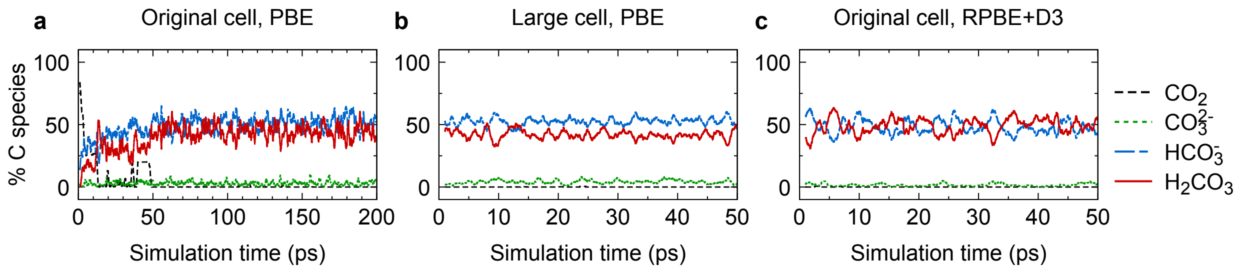

Supplementary Fig. 4. The 1 ps window average of the mole percents of aqueous carbon species in the graphene confinement at ∼10 GPa and 1000 K. The initial mole fraction of CO_2 is 0.185. (**a**) There are 5 CO_2 and 22 H_2O molecules in one unit cell. The exchange-correlation functional is PBE. (**b**) There are 10 CO_2 and 44 H_2O molecules in one unit cell. The exchange-correlation functional is PBE. (**c**) There are 5 CO_2 and 22 H_2O molecules in one unit cell. The exchange-correlation functional is RPBE with the D3 van der Waals corrections [7].

Supplementary Table IV. Equilibrium mole percents of carbon species in total dissolved carbon obtained by a large simulation and van der Waals corrections. The graphene-confined solutions are at ∼10 GPa and 1000 K.

Initial molecules	5 CO_2 + 22 H_2O	10 CO_2 + 44 H_2O	5 CO_2 + 22 H_2O
Functional	PBE	PBE	RPBE+D3
CO_2	1.3 ± 0.9	0.0	0.0
CO_3^{2-}	3.1 ± 0.1	4.1 ± 0.2	1.7 ± 0.2
HCO_3^-	50.0 ± 1.0	52.5 ± 0.4	48.4 ± 0.8
H_2CO_3	42.7 ± 1.7	42.1 ± 0.5	48.8 ± 1.0
$H_3CO_3^+$	1.3 ± 0.1	1.3 ± 0.1	1.0 ± 0.1
Pyrocarbonate	1.6 ± 0.8	0.0	0.0

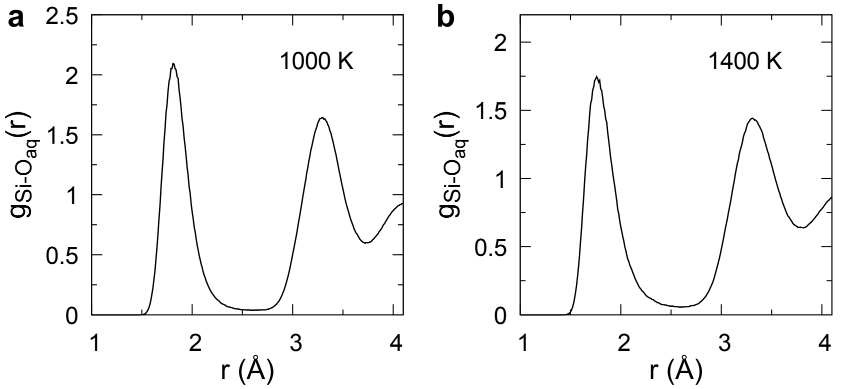

Supplementary Fig. 5. Radial distribution functions (RDFs) of the silicon atoms (Si) in the solid-liquid interface layer of stishovite versus the oxygen atoms (O_{aq}) in the solutions at (**a**) 1000 K and (**b**) 1400 K. The initial mole fraction of CO_2(aq) is 0.185. The oxygen atoms in the first peak of RDFs are considered as bonded to the silicon atoms in stishovite.

Supplementary Table V. Equilibrium mole percents of carbon species in total dissolved carbon obtained by varying the cutoff length of Si-O bonds. The stishovite-confined solutions are at ∼10 GPa and 1000 K.

Cutoff distance	2.6 Å	2.34 Å	2.86 Å
CO_2	10.5 ± 2.3	10.6 ± 2.3	10.0 ± 2.2
CO_3^{2-}	0.7 ± 0.1	0.8 ± 0.1	0.7 ± 0.1
HCO_3^-	18.2 ± 1.8	18.5 ± 1.8	17.9 ± 1.8
H_2CO_3	21.6 ± 2.7	21.8 ± 2.8	21.1 ± 2.6
$H_3CO_3^+$	0.5 ± 0.1	0.5 ± 0.1	0.5 ± 0.1
Pyrocarbonate	0.0	0.0	0.0
Surface groups	48.5 ± 2.1	47.9 ± 2.1	49.7 ± 2.2

Supplementary Table VI. Equilibrium mole percents of carbon species in total dissolved carbon obtained by varying δ. δ is defined as the difference between the third and second nearest C-O distances. The stishovite-confined solutions are at ∼10 GPa and 1000 K.

δ	0.4 Å	0.36 Å	0.44 Å
CO_2	10.5 ± 2.3	10.5 ± 2.3	10.5 ± 2.3
CO_3^{2-}	0.7 ± 0.1	0.7 ± 0.1	0.7 ± 0.1
HCO_3^-	18.2 ± 1.8	18.2 ± 1.8	18.2 ± 1.8
H_2CO_3	21.6 ± 2.7	21.6 ± 2.7	21.6 ± 2.7
$H_3CO_3^+$	0.5 ± 0.1	0.5 ± 0.1	0.5 ± 0.1
Pyrocarbonate	0.0	0.0	0.0
Surface groups	48.5 ± 2.1	48.5 ± 2.1	48.5 ± 2.2

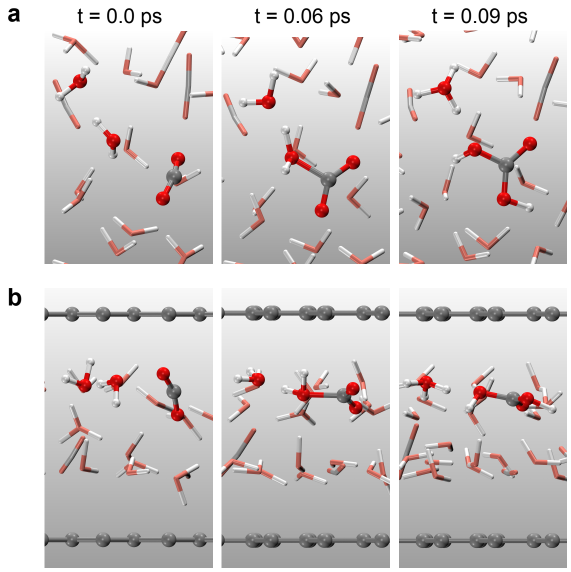

Supplementary Fig. 6. Reaction between water and CO_2(aq) under graphene nanoconfinement at $\sim$10 GPa and 1000 K. (**a**) Top view; (**b**) Side view. Carbon atoms are colored gray, oxygen atoms are colored red, and hydrogen atoms are colored white.

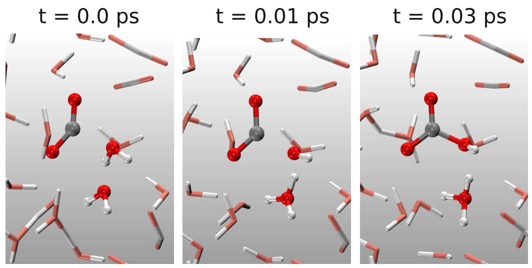

Supplementary Fig. 7. Snapshots of the reaction between OH^- and CO_2(aq) under graphene confinement at $\sim$10 GPa and 1000 K. The color scheme is the same as in Supplementary Fig. 6.

Supplementary Table VII. Average numbers of the OH^- and H_3O^+ ions in one unit cell per each AIMD snapshot under nanoconfinement. The initial mole fraction of CO_2 is 0.185 and the pressure is ∼10 GPa. Uncertainties are obtained using the blocking method [12].

T	Confinement	N_{OH^-}	$N_{H_3O^+}$
1000 K	Graphene	0.17 ± 0.01	2.96 ± 0.03
	Stishovite	0.04 ± 0.002	1.09 ± 0.03
1400 K	Graphene	0.23 ± 0.03	2.03 ± 0.05
	Stishovite	0.06 ± 0.004	0.82 ± 0.04

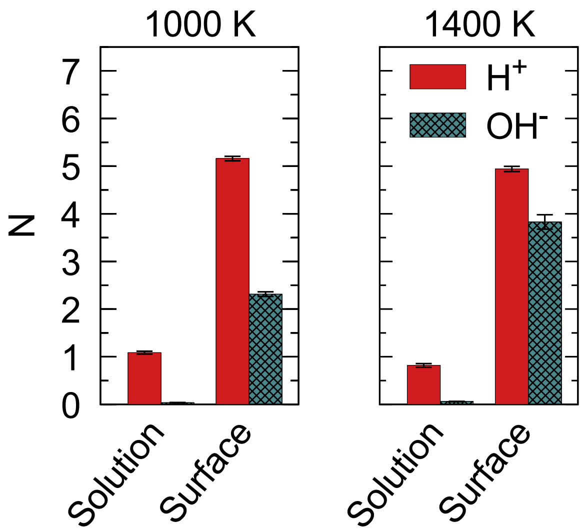

Supplementary Fig. 8. Average number (N) of H^+ and OH^- ions dissolved in the solutions and adsorbed at the stishovite surface in one unit cell per each AIMD snapshot. The initial mole fraction of CO_2(aq) is 0.185. The pressure is ∼10 GPa, and the temperature is 1000 K (left) and 1400 K (right).

SUPPLEMENTARY REFERENCES

[1] Ma, J. *et al.* Adsorption and diffusion of water on graphene from first principles. *Phys. Rev. B* **84**, 033402 (2011).

[2] Koga, K., Tanaka, H. & Zeng, X. First-order transition in confined water between high-density liquid and low-density amorphous phases. *Nature* **408**, 564–567 (2000).

[3] Chen, J., Schusteritsch, G., Pickard, C. J., Salzmann, C. G. & Michaelides, A. Two dimensional ice from first principles: Structures and phase transitions. *Phys. Rev. Lett.* **116**, 025501 (2016).

[4] Muñoz-Santiburcio, D. & Marx, D. Nanoconfinement in slit pores enhances water self-dissociation. *Phys. Rev. Lett.* **119**, 056002 (2017).

[5] Brandenburg, J. G. *et al.* Physisorption of water on graphene: Subchemical accuracy from many-body electronic structure methods. *J. Phys. Chem. Lett.* **10**, 358–368 (2019).

[6] Takeuchi, K. *et al.* Adsorption of CO_2 on graphene: A combined TPD, XPS, and vdW-DF study. *J. Phys. Chem. C* **121**, 2807–2814 (2017).

[7] Grimme, S., Antony, J., Ehrlich, S. & Krieg, H. A consistent and accurate ab initio parametrization of density functional dispersion correction (DFT-D) for the 94 elements H-Pu. *J. Chem. Phys.* **132**, 154104 (2010).

[8] Hamann, D. Optimized norm-conserving vanderbilt pseudopotentials. *Phys. Rev. B* **88**, 085117 (2013).

[9] Schlipf, M. & Gygi, F. Optimization algorithm for the generation of oncv pseudopotentials. *Comput. Phys. Commun.* **196**, 36–44 (2015).

[10] Feya, O. D. *et al.* Tetrahedral honeycomb surface reconstructions of quartz, cristobalite and stishovite. *Sci. Rep.* **8**, 11947 (2018).

[11] Stolte, N. & Pan, D. Large presence of carbonic acid in CO_2-rich aqueous fluids under Earth's mantle conditions. *J. Phys. Chem. Lett.* **10**, 5135–5141 (2019).

[12] Flyvbjerg, H. & Petersen, H. G. Error estimates on averages of correlated data. *J. Chem. Phys.* **91**, 461–466 (1989).
